# Functional Foods, a Hope to Delay Muscle Dystrophy Progression: A Potential Role for Omega Fatty Acids

**DOI:** 10.3390/nu17061039

**Published:** 2025-03-15

**Authors:** Ilaria Versari, Alberto Bavelloni, Mirko Traversari, Sabrina Burattini, Michela Battistelli, Pietro Gobbi, Irene Faenza, Sara Salucci

**Affiliations:** 1 Department of Biomedical and NeuroMotor Sciences (DIBINEM), University of Bologna, 40126 Bologna, Italy; ilaria.versari4@unibo.it (I.V.); irene.faenza2@unibo.it (I.F.); 2 Laboratory of Experimental Oncology, Istituto di Ricovero e Cura a Carattere Scientifico (IRCCS), Istituto Ortopedico Rizzoli, 40136 Bologna, Italy; alberto.bavelloni@ior.it; 3 Department of Medical and Surgical Sciences (DIMEC), University of Bologna, 40126 Bologna, Italy; mirko.traversari2@unibo.it; 4 Department of Biomolecular Sciences (DiSB), Urbino University Carlo Bo, 61029 Urbino, Italy; sabrina.burattini@uniurb.it (S.B.); michela.battistelli@uniurb.it (M.B.); pietro.gobbi@uniurb.it (P.G.)

**Keywords:** muscle dystrophy, fibrosis, inflammation, oxidative stress, omega-3 fatty acids, omega-6 fatty acids

## Abstract

Functional foods, thanks to their basic nutritional properties, can have physiological benefits and can alleviate the symptoms of many chronic diseases. They contain active components deriving either from plant or animal sources, and they show anti-inflammatory, cardiotonic, and antioxidant pharmacological activities that could be useful in preventing oxidative damage and inflammatory processes in a variety of disorders. There is evidence from in vitro, in vivo, and clinical observational studies that some compounds have significant effects in modulating the muscular dystrophy phenotype, which is characterized by fibrosis, myofiber necrotic cell death, inflammation, oxidative stress, and dysfunctional mitochondria. This review involves collecting data from the main medical databases and detailing the key features involved in muscular dystrophy progression and the relevance of fatty-acid compounds as diet supplements in the management of the disease. Omega fatty acids improve the dystrophic phenotype in terms of fibrosis and inflammation reduction, stimulating mitochondrial activity and antioxidant systems. Omega fatty acids could play a crucial role as food supplementation to delay dystrophy progression. This overview appears extremely relevant for researchers who are studying these molecules as valid alternatives to glucocorticoids, that today remain the only recognized pharmacological cure for dystrophic patients.

## 1. Muscular Dystrophies

Muscular dystrophies (MDs) are hereditary disorders characterized by inadequate or missing glycoproteins in the plasma membrane of muscles [1]. MDs are caused by mutations in more than 40 genes with distinct pathogenic mechanisms and clinical manifestations [2], defining several variations of the disease. Some of them lead to more rapid muscle wasting, physical disability, or reduced lifespan. Table 1 shows the main dystrophy diseases, classified based on the age of onset, principal pattern of muscle involvement, and other clinical features [3,4,5,6,7,8,9,10].

In general, MD affects the muscles, in particular the muscles of the lower limbs, such as gracilis, semimembranosus, semitendinosus, and sartorius. Dystrophy complications include scoliosis or lordosis with a disabling curvature of the spine that leads to breathing difficulties and cardiomyopathy [11]. Muscle loss and weakness, primarily due to genetic alterations [12,13], may also be the consequence of an imbalance in protein synthesis and breakdown caused by skeletal muscle inactivity [14,15,16,17,18] and inflammation processes [19,20,21].

To date, there is no effective cure for MD, even though several therapies have been developed, including genetic, cellular, and pharmacological approaches [22]. Innovative therapeutic treatments, such as nanomedicine for drug repurposing [23], are currently being developed but will not be available in the near future. Glucocorticoid treatment remains the standard care in MD, since it can delay disease progression and improve muscle strength. However, the use of glucocorticoid is also associated with significant adverse consequences including adrenal suppression, growth impairment, poor bone health and metabolic syndrome [23].

Despite the rise in synthetic drugs, natural products continue to exert a crucial role due to their unique chemical structures and biological activities. In fact, most natural products can interact with molecules involved in key intracellular pathways correlated to MD progression.

Recently, the attention has been focused on functional foods [24], which exert anti-inflammatory or antioxidant effects, providing health or medical benefits against muscular disorders. Among them, resveratrol, coenzyme Q10, curcumin, and various polyphenols [25,26] are worth mentioning, as well as essential fatty acids for their potential role in alleviating dystrophic disease progression.

## 2. Common Features of MD

MD has common histopathological and molecular features representing the consequence of disease progression. The main shared trait is fibrotic tissue deposition, triggered by the degeneration and cell death of muscle fibers, chronic inflammation, and elevated oxidative stress; the latter factors promote the infiltration of inflammatory cells and the oxidation of lipids and proteins [27].

### 2.1. Fibrosis

Fibrosis is considered the primary common feature of MD, characterized by the replacement of functional muscle with connective tissue. Fibrosis is the consequence of genetic mutations of proteins (e.g., dystrophin) that link myofibers to the ECM [28,29]. The process of fibrosis is favored by degeneration and regeneration cycles with an incomplete ECM remodeling. The latter event induces an accumulation of matrix components, leading to muscle instability and progressive contractile tissue loss [30,31,32]. Fibrogenic signals depend on transforming growth factor-beta (TGFβ), whose levels are increased in MD, which leads to high collagen production.

**Figure 1:**
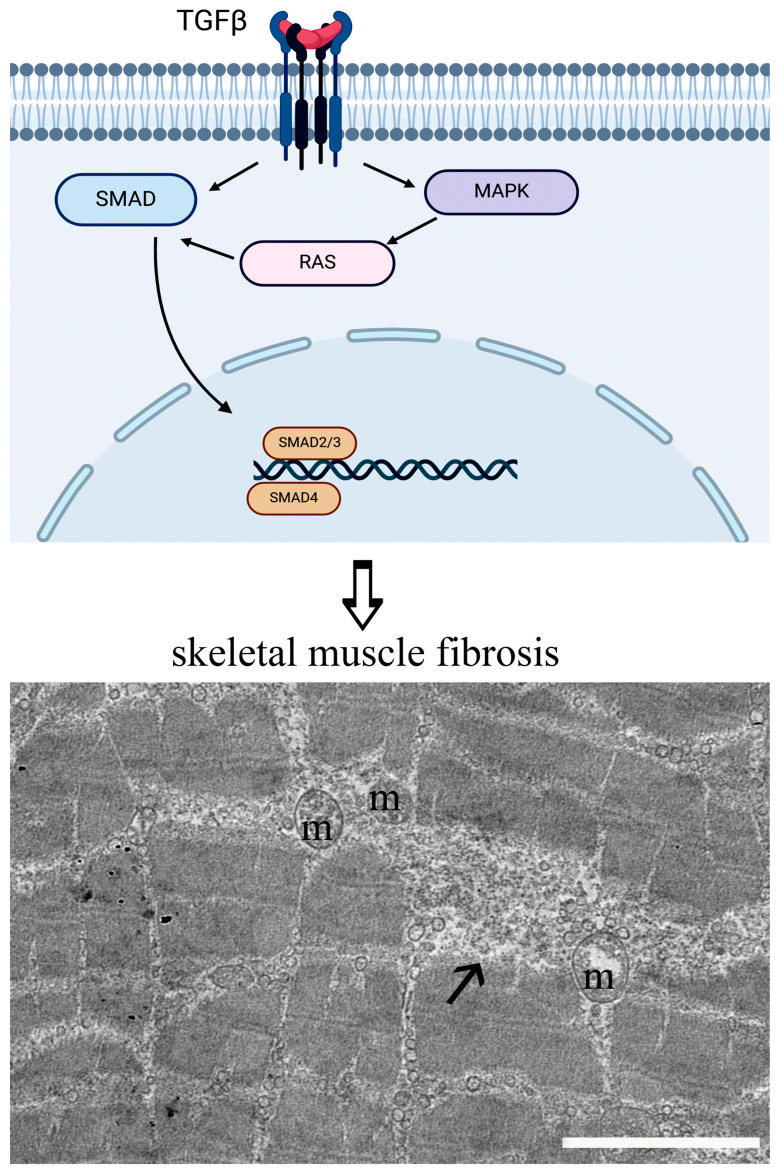
A schematic representation of the TGFβ fibrotic pathway. The TEM micrograph shows fibrosis (arrow) in dystrophic skeletal muscle tissue. Fibrotic tissue appears intercalated between the myofibrils which consist of repeating units of contractile actin and myosin protein filaments. Z-bands that delineate the lateral borders of sarcomeres are no longer preserved. Damaged mitochondria (m) can also be observed. Bar: 2 µm.

The synthesis of profibrotic factors (Figure 1), including TGFβ, occurs at the nuclear level as a result of a positive feedback loop involving the SMAD complex and, indirectly, MAPK signaling. This process also integrates signals from the renin–angiotensin system (RAS), a downstream pathway of TGFβ [33]. Specific fibrotic pathways in skeletal muscle are regulated by myostatin, which promotes the proliferation of muscle fibroblasts together with the production of extracellular matrix proteins, and its targets converge onto SMAD signaling [34]. TGFβ is also involved in promoting the proliferation of fibro-adipogenic progenitor cells (FAPs), mesenchymal stem cells that play a crucial role in muscle health and regeneration. The dysregulation of FAP activity may lead to atrophy and fibrosis [35,36]. In MD, FAPs’ proliferation and TGFβ are permanently activated, and macrophages constantly release growth factors with a consequent expansion of the fibrotic tissue [37]. Furthermore, FAP cells express factors involved in degeneration and regeneration processes. Among them, PDGFRα is a tyrosin kinase receptor that, when phosphorylated, triggers the activation of RAS, MAPK, PI3K, or PLC-γ pathways, which control proliferation, cell differentiation, apoptosis inhibition, the mobilization of intracellular calcium, and cell motility [38]. Recent studies showed that treating murine models of DMD with tyrosine kinase inhibitors results in the downregulation of PDGFRα with a reduction in muscle fibrosis and an improvement in muscle function [39,40,41]. Incorporating micronutrients through a balanced diet rich in fruits, vegetables, whole grains, lean proteins, and healthy fats can enhance FAPs’ function and support muscle regeneration, especially in older adults or individuals experiencing muscle-wasting conditions [42].

### 2.2. Inflammation and Oxidative Stress

**Figure 2:**
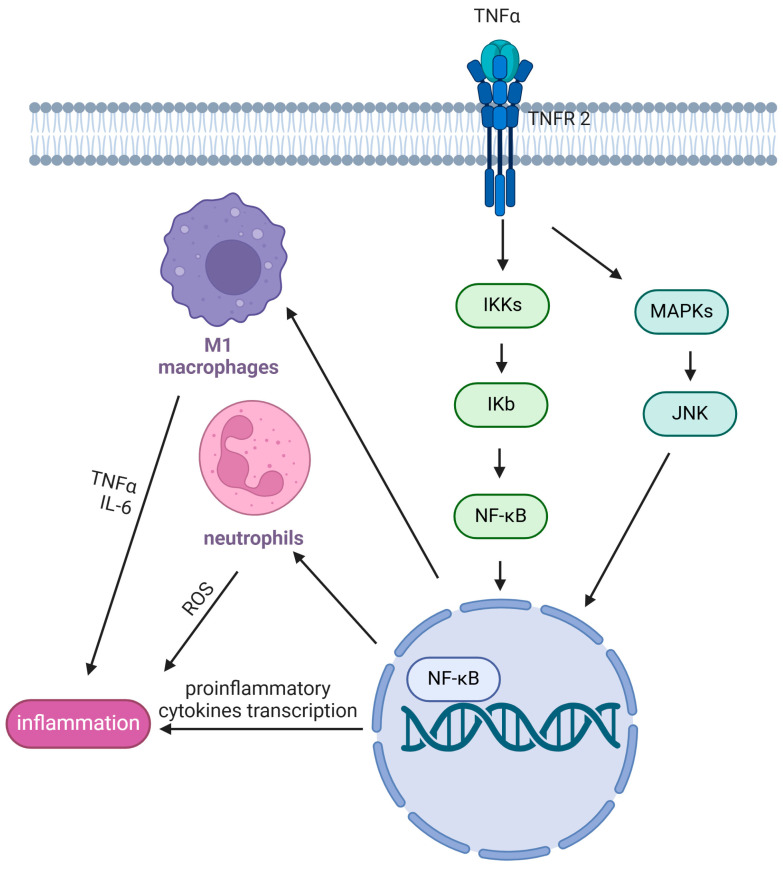
A schematic representation of the NF-κB inflammatory pathway in MD.

Inflammation is crucial for proper skeletal muscle repair but paradoxically also drives MD pathology [43,44]. The main inflammatory pathway upregulated in MD is the nuclear factor kappa B (NF-κB) pathway (Figure 2), which involves c-Jun NH2-terminal kinase (JNK), IκB kinases (IKKs), mitogen-activated kinases (MAPKs), TNF-α, and interleukin 6 (IL-6) activations [45].

Several studies demonstrated that NF-κB inhibition effectively reduces inflammation and attenuates dystrophic muscle pathology [46,47] largely due to the involvement of PGC-1α, the key regulator of mitochondrial function [48,49,50]. Macrophages and neutrophils are the main immune-cell types involved in MD injury and when they persist in muscle tissue, they induce an alteration of the microenvironment, which is no longer suitable for proper muscle regeneration (Figure 2). In healthy muscles, pro-inflammatory macrophages (known as M1) are essential for phagocytosis processes, ECM remodeling, and myogenesis. On the other hand, anti-inflammatory macrophages (known as M2) appear crucial for myofiber regeneration [51]. In MD, continuous M2 activation contributes to excessive ECM accumulation and a dysfunctional population of muscle progenitor cells, hindering muscle regeneration [52]. At the same time, M1 cells promote myonecrosis and excessive inflammation [53,54]. In addition, in mdx mice, neutrophils can activate a defense response and simultaneously release high levels of free radicals, leading to inflammation and increased oxidative stress [55,56].

High levels of pro-inflammatory cytokines stimulate the production of pro-oxidant radicals, which contribute to muscle damage [44] by affecting mitochondria [57,58]. The increased production of reactive oxygen species (ROS), caused by the imbalance between ROS generation and antioxidant defense, can alter skeletal muscles and exacerbate MD symptoms [59]. MD are characterized by a deficiency in antioxidant enzymes [59], resulting from ongoing damage caused by a lack of dystrophin and improper inflammatory response [60,61,62,63,64].

All of these pathways may be modulated by polyunsaturated fatty acids, which are precursors of potent lipid mediators known as eicosanoids with important roles in the regulation of inflammation [65].

## 3. Omega Fatty Acids

Fatty acids are key components of cell-membrane phospholipids and play essential roles in regulating various functions, including cell metabolism and signaling [66]. Fatty acids usually contain an even number of carbon atoms, and are generally unbranched. They can be classified by the presence and number of carbon-to-carbon double bonds and are officially numbered from the carboxylic carbon (-COOH), also known as carbon alpha. According to the position of the double bond, fatty acids can be divided into omega-3, omega-6, and omega-9, the latter of which is a group of non-essential unsaturated compounds. The main omega-9 component is oleic acid, which is considered an alternative to saturated animal fats and also an anti-inflammatory molecule; however, to date, there are controversial scientific results on its biological value [67].

**Figure 3:**
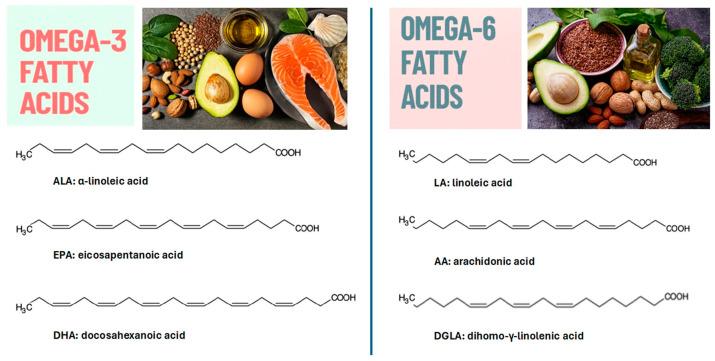
The chemical structures of omega-3 and omega-6. Omega-3 can be found in walnuts, flaxseeds, sardines, salmon, spinach, eggs, brussels sprouts, and chia seeds. Omega-6 can be found in flaxseed oil, seeds, nuts, grapeseed oil, and other vegetable oils.

The most studied polyunsaturated fatty acids (PUFAs), which have two or more double bonds, are classified into two main subgroups: *n*-6 long chain PUFAs (*n*-6 LC-PUFAs) and *n*-3 long chain PUFAs (*n*-3 LC-PUFAs) (Figure 2), which are commonly referred to as omega-6 and omega-3, respectively [68,69]. PUFAs are essential nutrients that have to be introduced from the diet, and their intake appears necessary for modulating numerous pleiotropic cell functions. PUFAs can act as lipid mediators and building blocks of membrane lipids. PUFAs’ lipid mediators are considered strong antioxidants, able to control and modulate inflammatory processes [65,70]. The crucial PUFAs for human health are α-linolenic acid (ALA, C18:3*n*-3), eicosapentaenoic acid (EPA, C20:5*n*-3), and docosahexaenoic acid (DHA, C22:6*n*-3) from the *n*-3 family, and linoleic acid (LA, C18:2*n*-6), dihomo-γ-linolenic acid (DGLA, 20:3*n*-6), and arachidonic acid (AA, C20:4*n*-6) from the *n*-6 family (Figure 3) [71,72,73].

Omega fatty acids are synthesized in plants and can be found in several plant-based foods, such as seeds, nuts, and plant oils. In general, PUFAs’ deficiency, mainly correlated with a deficit of LA and ALA, results in several pathologies including dermatitis, renal hypertension, mitochondrial activity disorders, cardiovascular diseases, type 2 diabetes, impaired brain development, arthritis, depression, and decreased resistance to infection [74]. LA and ALA are therefore essential compounds for cell growth and repair, and they also play a crucial role in the production of other fatty acids [75,76]. Furthermore, omega-6 PUFAs are also involved in regulation of inflammation, immunity, blood-vessel homeostasis, and platelet aggregation [77,78,79].

On the other hand, omega-3 molecules can inhibit inflammation, platelet aggregation, and stimulate vasodilation [80,81]. LA, an omega-6 PUFA, acts mainly through lowering LDL-cholesterol, while both EPA and DHA (omega-3 PUFAs) lower triglycerides, promote blood flow, improve cardiac and vascular function, and control thrombosis and inflammation [74]. In particular, DHA is a structural constituent of membranes in the central nervous system, and its intake can promote brain development and function [82].

Epidemiological studies and clinical trials enhanced the correlation between omega-3 beneficial consumption and the reduction in inflammatory symptoms. In particular, EPA and DHA are the main omega-3 PUFA compounds with an anti-inflammatory profile [77,83,84].

Therefore, since the antioxidant and anti-inflammatory properties of omega fatty acids are recognized to decrease disease risk and severity [74], they may be considered potential therapeutics, acting as lipid mediators, in conditions such as muscular dystrophies that are marked by excessive inflammation and oxidative stress. In fact, lipid mediators can modulate this inflammatory response, potentially mitigating muscle loss associated with dystrophic conditions [85].

## 4. Omega Fatty Acids and Dystrophies

While gene therapies for exon skipping and treatments targeting molecular pathways involved in dystrophy pathology have been developed, their success has been limited. Therefore, glucocorticoids remain the only treatment capable of delaying disease progression [86,87]. However, their well-known adverse effects in long-term therapy have prompted researchers to find alternatives. One such adjuvant therapy is polyunsaturated fatty acids, which aim at reducing inflammation in patients with MD. Their efficacy in reducing inflammation and fibrosis across various disorders has been demonstrated, leading to their recent incorporation into the treatment of several inflammatory diseases, including MD [74,88,89,90]. Several experimental studies in dystrophic mice, as well as epidemiological research on dystrophic patients, demonstrated the potent ability of omega-fatty-acid supplementation to delay dystrophy progression, showing an effect comparable to that of glucocorticoids in some experiments.

Initial studies conducted on dystrophic animal models demonstrated that the administration of omega-3 significantly decreased muscle inflammation and enhanced muscle regeneration [91,92]. The omega-3 effect was evaluated in the diaphragm and quadriceps femoris of mdx mice through histopathological analyses. Hematoxylin–eosin staining showed, in muscles of mdx mice supplemented with omega-3, reduced fibrosis and the presence of new muscle fibers, evidenced by an elevated number of centrally nucleated fibers [93]. Moreover, in the same animals, omega-3 treatment reduced plasma levels of pro-inflammatory (TNF-α, INF-γ, IL-6) and pro-fibrotic (IL-13 and TGF-β) interleukins and increased plasma levels of IL-10. Therefore, omega-3 supplementation in dystrophic mice reduced myonecrosis, fibrosis and inflammation, as demonstrated by several authors [91,94,95].

These findings have been supported by other experiments. For instance, Tripodi and colleagues analyzed the effects of a mixture of omega-3 fatty acids and flavonoids in mdx mice. This compound, called FLAVOmega β, favored the preservation of muscle mass and stimulated fiber regeneration when administered to dystrophic animals, evidenced by an increase in satellite cell numbers and myogenic protein levels. Histological analyses reveal that treatment with FLAVOmega β leads to fewer inflammatory cell infiltrates, reduced fibrosis, and an improved muscle environment, contributing to better overall muscle health and performance in dystrophic mice. The formulation affects inflammation by reducing the levels of pro-inflammatory cytokines, such as TNF-α and IL-6 [96]. Carotenuto and colleagues demonstrated a reduction in fibrosis in dystrophic animals by supplementing their diet with flaxseeds, which are among the richest sources of omega-3 fatty acids. This dietary intervention notably enhanced the animals’ lifespan [97]. In particular, dystrophic hamsters consuming flaxseeds showed a reduction in apoptotic cells and CD45 cells in addition to a downregulation of TNF levels. Moreover, flaxseed administration enhanced the number of cells expressing myogenesis markers, such as myogenin and caveolin-3. These beneficial effects of flaxseeds on the modulation of the inflammatory microenvironment and in myogenesis can be attributable mainly to the ALA content [98]. Omega-3 treatment administrated in the early stage of dystrophy reduced myonecrosis and inflammation as well as pro-inflammatory markers [88,94], and the ability of omega-3 to delay pathology progression was also demonstrated in the late stage of the disease in mdx diaphragm. Diaphragm fibers during the late stage of dystrophy showed alterations similar to those described in human disease. In this context, Fogagnolo and colleagues [94] demonstrated that omega-3 supplementation delayed fibrosis in dystrophic diaphragm by reestablishing the balance between metalloproteinase-9 (MMP-9) and tissue inhibitors of metalloproteases (TIMPs), a crucial factor for maintaining tissue integrity [99].

Furthermore, in late-stage mdx mice, omega-3 was able to reduce markers related to oxidative stress and restore abnormal calcium homeostasis by lowering levels of activated transient receptor canonical protein 1 (TRPC1, strictly correlated to a high calcium influx in dystrophic mice) and increasing calsequestrin activity [100]. This latter finding appears very interesting, since it is known that calcium dysregulation and oxidative stress contribute to disease progression [101].

Moreover, omega-3 treatment of late-stage dystrophic mdx mice can positively modulate some metabolites such as leucine, isoleucine, and valine, all linked to muscle regeneration [100]. In addition to its capacity to protect muscle fibers from degeneration, omega-3 has been shown to play a crucial role in promoting regeneration and activating satellite cells [95]. Indeed, omega-3 treatment increased the number of centrally nucleated fibers in the quadriceps of mdx mice [101], along with a rise in embryonic myosin heavy-chain positive fibers and an enhanced satellite-cell population [95].

Clinical studies on the usage of omega-3 in dystrophic patients were published starting from 2017. The first double-blind, randomized controlled trial demonstrated that six months of treatment with omega-3 fatty-acid long chain reduced chronic inflammation in DMD patients with a downregulation of pro-inflammatory cytokines in serum and of NF-κB expression in leukocytes [102]. Another single-center randomized double-blind placebo-controlled study assessed the efficacy of dietary supplementation of flavonoid- and omega3-based natural supplements (FLAVOMEGA) in DMD patients. Researchers evaluated the endurance (6 min walking distance) in walking patients and measured the muscle strength of upper and lower limbs through a dynamometer in both walking and wheelchair patients. The study demonstrated a statistically significant effect of FLAVOMEGA treatment in increasing muscle performance in DMD compared to the untreated subjects [103,104].

More recently, attention has been focused on some mediators originating from the conversion of omega-3 fatty acids by enzymes such as 5-lipoxygenase and 15-lipoxygenase. Among these mediators, Resolvin exhibited potent anti-inflammatory effects, reducing fibrosis, increasing muscle-fiber size, and boosting the number of myogenic cells in DMD mice receiving this treatment. To date, Resolvin is considered a pro-resolving mediator in the inflammation process, due to its ability to interact as an agonist with G Protein-Coupled Receptor 18 (GPR18), known to exert a crucial role in inflammation modulation [105]. The Resolvin compound exhibits a profile that closely resembles that of glucocorticoids, such as prednisolone; however, unlike glucocorticoids, resolvin is also capable of stimulating myogenesis [106,107]. This finding aligns with evidence suggesting that the progression of Duchenne muscular dystrophy (DMD) is driven not only by muscle degeneration but also by impaired muscle regeneration. However, Resolvin does have certain limitations; for instance, this bioactive lipid is not well absorbed in the gastrointestinal tract and is readily degraded by enzymes in vivo [108]. Therefore, its molecular structure may be considered as a starting point to develop more stable synthetic analogs that are able to improve muscle function in DMD [109].

In addition to the positive effects in the modulation of inflammation, fibrosis, and regeneration, omega-3 polyunsaturated fatty acids can be considered immunomodulators. In fact, in dystrophic muscles of mdx mice, they reduced the activity of pro-inflammatory macrophages by promoting the activity of anti-inflammatory ones [88,92,94,110]. Rodriguez and colleagues [102] demonstrated that DMD patients treated with omega-3 showed a downregulation of pro-inflammatory genes on the mRNA level. Furthermore, this decrease has been observed in serum, together with an upregulation of anti-inflammatory cytokines [111]. Recent observations indicate that monocyte subpopulations in patients with DMD are altered, accompanied by elevated levels of circulating pro-inflammatory cytokines such as IL-1β, IL-6, and TNF-α. Treatment with omega-3 fatty acids has shown a reduction in the number of pro-inflammatory cells and an increase in the presence of anti-inflammatory cells, as evidenced by muscle biopsy evaluation from these patients [90].

Ultimately, randomized studies have demonstrated that omega-3 fatty acids can effectively slow the decline of skeletal muscle mass while promoting a reduction in fat mass among patients with dystrophic conditions. This beneficial effect of omega-3 fatty acids in the prevention of adipose tissue hyperplasia and hypertrophy had already been demonstrated in rodents and human patients with metabolic syndrome [112].

Therefore, omega-3 treatment delays complications such as hyperinsulinemia and insulin resistance, correlated with excessive fat mass in MD subjects [94,111,112,113]. As a matter of fact, chronic inflammation, fibrosis, and impaired muscle function are known characteristics of MD that can be exacerbated by obesity [112].

There is limited information available regarding the effects of omega-6 fatty acids on delaying MD, and most articles are focused on its main component, arachidonic acid (AA). AA is a precursor of several metabolites like prostaglandins, leukotrienes, thromboxane, and lipoxins, involved in the control of skeletal muscle development and growth by regulating proliferation, differentiation, migration, fusion, and the survival of myoblasts [114]. However, the activity of AA and of its metabolites appears controversial. It is known that some metabolites have a role in disease progression and are involved in muscle-fiber degeneration and regeneration. For this reason, some potential strategies to prevent dystrophy progression involve the inhibition of the pro-inflammatory AA metabolites (e.g., prostaglandins) or the enhancement of the anti-inflammatory ones (e.g., lipoxins) [115]. In the context of MD, it has been shown that levels of arachidonic acid (AA) are diminished in both muscle tissues and erythrocytes of patients with DMD. This reduction disrupts calcium homeostasis and contributes to oxidative stress and peroxidation events [115]. Omega-9 fatty acids may play a potential role in inflammatory disorders due to their beneficial properties, but there are no data regarding their role in MD. Only one in vitro study on skeletal muscle cells demonstrated that omega-9 fatty acids control lipid peroxidation involving PGC1α activation [116].

## 5. Conclusions

The available data suggest that fatty acids, particularly omega-3, may regulate the inflammatory environment in MD by enhancing the activity of anti-inflammatory cytokines and macrophages. Omega fatty acids provide a multidimensional approach to supporting muscle health by reducing inflammation, fibrosis, and oxidative stress while also promoting regeneration and maintaining calcium balance. These benefits can collectively aid in delaying the progression of MD and improving the quality of life of affected individuals. The effects of omega-6 and -9 activity on MD are poorly described, due to the controversial functions of its metabolites, which may both exacerbate the disease and slow its progression. There is a need to identify specific molecular targets for omega-6 fatty acids that may help mitigate muscle loss and function in dystrophic cases. Understanding how these nutrients interact at a molecular level could lead to more precise dietary recommendations. This review suggests that omega fatty acids, particularly omega-3, may serve as promising alternatives or adjuncts to traditional treatments for MD, especially glucocorticoids. Currently, glucocorticoids are the only recognized pharmacological treatment available to delay the progression of MD, but they are associated with significant long-term adverse effects. This review highlights that omega-3 fatty acids may provide similar benefits in terms of delaying disease progression while potentially having fewer side effects, thus representing a safer alternative. Clinical studies cited in this review demonstrate that omega-3 fatty acids can reduce chronic inflammation and improve muscle strength and endurance over traditional treatments. For instance, omega-3 supplementation has shown significant effects on reducing markers of inflammation in patients with DMD [111].

This review underscores the beneficial role of omega-3 supplementation in preclinical dystrophic models and in human dystrophic patients. Omega-3 preserves muscle function by reducing fibrosis and oxidative stress, stimulating myogenesis, reestablishing calcium balance, improving the maintenance of muscle mass, and decreasing fat mass accumulation. Therefore, since nutrition is one of the major exogenous factors able to modulate cellular function, omega-3 dietary supplementation may represent a simple and available approach, in combination with other drugs, to reduce complications and symptoms of MD. Therefore, the potential beneficial effects of omega-3 in delaying MD progression should be evaluated in a large number of MD patients to validate its use as adjuvant therapy or in palliative care for improving quality of life. Understanding how these nutrients interact at a molecular level could lead to more precise dietary recommendations. As nutrition is a significant modifiable factor in disease management, future studies may explore personalized dietary interventions that tailor omega-fatty-acid intake according to individual patient needs, potentially leading to improved management strategies.

## Figures and Tables

**Table 1:** The features of the main dystrophy diseases.

MD Disease	Main Pathological Features	Age of Onset (Range)	Gender
**DMD**	Severe muscle weakness with frequent falls, difficulty walking, and wheelchair dependence	Childhood	Men
**Beker dystrophy**	progressive weakness and wasting	Slow progression 11–25 years	Men
**Myotonic muscular dystrophy**	Muscle loss, weakness, myotonia	Adults	Men and women
**Limb-Girdle Muscular Dystrophy**	muscle weakness of the upper arms, upper legs, shoulders, and hips	2–40 years	Men and women
**Fascioscapulohumeral Muscular Dystrophy**	Facial weakness, scapular winging, foot drop	15–30 years	Men and women
**Emery–Dreifuss Muscular Dystrophy**	Joint contractures, muscle weakness	adolescent	Men and Women
**Distal Muscular Dystrophy**	Distal muscle weakness	40–60 years	Men and women
**Oculopharyngeal Muscular Dystrophy**	Muscle weakness of eyelids, face, and throat	40–50 years	Men and women
**Collagen Type VI-Related Disorders**	Low muscle tone, with joint laxity, contractures of the arms or legs, and decreased flexibility of the spine.	Childhood, sometimes in adolescence and adulthood.	Men
